# Fasting vs Nonfasting, Dose-adjusted Levothyroxine Ingestion in Hypothyroidism: A Randomized Clinical Trial

**DOI:** 10.1210/clinem/dgaf686

**Published:** 2025-12-23

**Authors:** Jeresa I A Willems, Daan J L van Twist, Floris Helmich, Thijs Sluiter, Marco Medici, Robin P Peeters, Roderick F A Tummers-de Lind van Wijngaarden

**Affiliations:** Department of Internal Medicine, Zuyd Thyroid Center, Zuyderland Medical Center, 6162 BG Sittard-Geleen, The Netherlands; Department of Internal Medicine, Zuyd Thyroid Center, Zuyderland Medical Center, 6162 BG Sittard-Geleen, The Netherlands; Department of Clinical Chemistry and Hematology, Zuyderland Medical Center, 6162 BG Sittard-Geleen, The Netherlands; Department of Internal Medicine, Zuyd Thyroid Center, Zuyderland Medical Center, 6162 BG Sittard-Geleen, The Netherlands; Academic Center for Thyroid Diseases, Department of Internal Medicine, Erasmus Medical Center, 3015 GD Rotterdam, The Netherlands; Academic Center for Thyroid Diseases, Department of Internal Medicine, Erasmus Medical Center, 3015 GD Rotterdam, The Netherlands; Department of Internal Medicine, Zuyd Thyroid Center, Zuyderland Medical Center, 6162 BG Sittard-Geleen, The Netherlands

**Keywords:** levothyroxine, hypothyroidism, breakfast, fasting ingestion

## Abstract

**Introduction:**

Levothyroxine (LT4) is recommended for intake in a fasting state to optimize absorption. However, fasting intake is often burdensome and may reduce adherence. In a previous questionnaire study, we observed a strong patient preference for taking LT4 with breakfast. Therefore, we conducted a randomized controlled trial to evaluate whether nonfasting LT4 intake—accompanied by a 15% dose increase—could maintain TSH stability compared to fasting LT4 intake.

**Methods:**

Adults with well-controlled hypothyroidism were randomized to fasting or dose-adjusted, breakfast LT4 intake. TSH, free T4, and total T3 were measured every 6 weeks, followed by LT4 dose adjustment if needed. The primary outcome was TSH stability, defined as 2 consecutive values within the reference range and a maximum ±1 mIU/L change from baseline. Patients were followed until TSH stability was reached, with a maximum of 24 weeks. After the initial study period, patients in the fasting group were invited to cross over to nonfasting intake, with similar follow-up.

**Results:**

Eighty-eight patients (80.7% female, median age 62y [interquartile range: 49-69]) were randomized to fasting (n = 43) or breakfast intake (n = 45). TSH stability was comparable between groups: 74.4% [95% confidence interval (CI): 61.0-88.0%] in the fasting vs 73.3% (95%CI: 60.0-87.0%) in the breakfast group (*P* = not significant). Similar findings were observed in the crossover group. The breakfast group reported greater improvement in self-reported well-being (33.3% vs 16.3%, *P* = .07) and a stronger preference for nonfasting intake (76.2% vs 44.2%, *P* < .001). By the end of the study, 88.9% chose to continue nonfasting intake.

**Conclusion:**

LT4 ingestion with breakfast with a 15% dose increase maintained TSH stability and improved patient well-being. Given the strong patient preference, this patient-centered approach may offer a viable alternative to fasting administration.

Levothyroxine (LT4) is one of the most prescribed drugs worldwide (1). Clinical guidelines recommend LT4 to be taken on an empty stomach—30 to 60 minutes before breakfast and separated from medication known to interfere with absorption—to prevent reduced gastrointestinal uptake (2-4). However, in a previous questionnaire study conducted by our research group, 50% of patients reported that delaying breakfast was burdensome (5). One-third admitted not consistently adhering to the recommended timing, and some reported regularly forgetting to take their medication due to the fasting requirement—both contributing to suboptimal LT4 therapy. Additionally, 25% of patients reported skipping breakfast altogether to meet the fasting condition.

Importantly, we observed a strong patient preference for taking LT4 with breakfast (5), a finding consistent with those of other studies (6-9). These observations raise the question of whether routine coingestion of LT4 with breakfast could be a viable alternative to fasting administration—provided that the expected reduction in absorption is offset by an appropriate dose adjustment.

Therefore, we conducted a randomized clinical trial comparing traditional fasting LT4 intake with LT4 intake alongside breakfast, accompanied by a 15% dose increase. We assessed whether this alternative regimen could maintain TSH stability and improve patient satisfaction among individuals with well-controlled hypothyroidism.

## Methods

### Study Design

This open-label, randomized clinical trial was conducted in Zuyderland Medical Center (The Netherlands). Ethical approval was granted by the institutional ethical review board (METCZ20230015), and the study was registered in the Dutch National Trial Registry (NL-OMON53296, https://onderzoekmetmensen.nl/nl/trial/53296). Written informed consent was obtained from all patients. Patient safety and benefit-risk evaluation were monitored by an independent external data committee.

### Patients

Patients with hypothyroidism, aged ≥ 18 years, using LT4 were eligible for inclusion if they met the following criteria: receiving a minimum LT4 dose of ≥ 1.0 mcg/kg, consuming breakfast on ≥ 5 days per week, and having 2 consecutive TSH levels within the assay-specific reference range before study participation. If one of the last two TSH measurements before enrollment was outside the reference range (0.27-4.20 mIU/L), the study was preceded by a run-in period of up to 12 weeks, during which repeat TSH measurements were allowed. Exclusion criteria were high-risk thyroid carcinoma, immunotherapy-induced hypothyroidism, central hypothyroidism, pregnancy or lactation, active malabsorption disorders (eg, inflammatory bowel disease or celiac disease), or debilitating chronic conditions with poor prognosis (eg, advanced heart failure, end-stage renal disease, or active malignancy).

### Randomization and Masking

Eligible patients were randomly assigned to either the fasting group or the breakfast group in a 1:1 ratio. This was an open-label trial; patients and investigators were aware of group allocation. The fasting group was instructed to take LT4 30 to 60 minutes before breakfast. The breakfast group was instructed to take LT4 with breakfast, with an empirically chosen 15% dose increase to compensate for the expected reduction in absorption of LT4 (10). Patients remained in their assigned group (fasting or breakfast) for the entire study period, taking LT4 according to the allocated regimen.

### Procedures

All patients remained on the same LT4 brand as before study participation, and no changes to the LT4 brand were permitted during the study. Adjusted LT4 doses were rounded to the nearest available whole tablet dose (ie, 75, 88, 100, 112, 125, 137, 150, 175, or 200 mcg for LT4 brands Euthyrox, Thyrofix, and Teva or to the nearest 12.5 mcg for patients using LT4 brand Thyrax). The LT4 dose was not adjusted for patients using the soft-gel formulation, as previous studies suggested that food and coffee do not affect absorption (11, 12). For patients already taking LT4 with breakfast, the dose remained unchanged when randomized to the breakfast group, while a 15% dose reduction was applied when randomized to the fasting group. The use of interfering drugs (including calcium-, iron-, or magnesium-containing supplements; vitamin supplements; potassium and phosphate binders; proton pump inhibitors; and H2-receptor antagonists) was allowed. However, patients were instructed to maintain a 4-hour interval between LT4 and these medications and to report any dose changes or new prescriptions. No restrictions were imposed on the content of breakfast.

Every 6 weeks, a follow-up visit with laboratory testing was scheduled. If TSH levels were outside the reference range, LT4 dose was adjusted according to a prespecified protocol. Patients were followed until TSH levels were within the reference range during 2 consecutive follow-up visits, up to a maximum of 24 weeks. Consequently, the duration of follow-up could be either 12, 18, or 24 weeks, depending on the TSH levels reached. If patients did not achieve TSH values within the reference range after a 24-week study period, this was considered as failure to achieve TSH stability with LT4 intake with breakfast, and these patients returned to traditional fasting LT4 intake. During follow-up visits patients were asked about potential thyroid-related symptoms and interfering factors (such as weight changes or intermittent illnesses). Breakfast content was recorded using breakfast diaries (Table S1) (13). At study completion, patient-reported outcome measures and patient-reported experience measures related to well-being and preferred LT4 ingestion regimen were assessed.

All patients in the fasting group who completed the study and achieved TSH stability at study completion were offered the option to crossover to nonfasting ingestion (hereafter referred to as the “crossover group”). Reasons for opting out of crossing over were collected. Additionally, patients in the crossover group completed the validated Dutch version of the ThyPRO-39 questionnaire (14) to evaluate the effect of switching from fasting to nonfasting ingestion on quality of life (QoL). The questionnaire includes 13 domains, each with 3 to 9 questions rated on a 0 to 4 Likert scale. Sum scores per QoL domain were transformed to a 0 to 100 scale.

### Outcomes

The primary outcome of this study was the proportion of patients achieving TSH stability in each group. TSH stability was defined as meeting both of the following criteria: (1) two consecutive TSH measurements remaining within the reference range and (2) a maximum change of 1 mIU/L (increase or decrease) from baseline. Secondary outcomes included self-reported well-being, preferred ingestion regimen, and the willingness to continue this LT4 regimen in the future. Reasons for changes in well-being were collected, allowing patients to report multiple options. For the crossover group, the same primary and secondary outcomes were assessed, along with additional evaluation of changes in QoL.

### Laboratory Testing

Blood samples were obtained every 6 weeks in a fasting state, before LT4 ingestion. TSH (reference range: 0.27-4.20 mIU/L) was measured using a sandwich electrochemiluminescence immunoassay (Elecsys® TSH), while free T4 (FT4, reference range: 11.0-22.0 pmol/L) and total T3 (TT3, reference range: 1.3-3.1 nmol/L) were measured using a competitive electrochemiluminescence immunoassay (Elecsys FT4 III and Elecsys T3, respectively). All measurements were performed on the Cobas Pro platform using the Cobas e analyzer (Roche, Basel, Switzerland).

### Statistical Analysis

In the primary analysis, we compared the fasting group with the breakfast group. Data from patients in the crossover group were analyzed separately to assess intraindividual variation in TSH levels. Categorical variables were expressed as frequencies and percentages, and continuous data were reported as means with SD or medians with interquartile range, as appropriate. For between-group comparisons, we used the chi-square test or Fisher's exact test for categorical variables and the independent sample *t*-test or Mann-Whitney U-test for continuous variables, as appropriate. Intraindividual comparisons in the crossover group were performed using the McNemar test for categorical variables and the paired-sample *t*-test or Wilcoxon signed-rank test for continuous variables. *P*-values < .05 were considered statistically significant. Sample size was calculated using G*Power software (version 3.1.9.7, Heinrich-Heine-Universität Düsseldorf, Germany). Assuming a clinically relevant 30% difference in TSH stability between both groups, 39 patients per group were required to achieve 80% power at a .05 significance level. Additional patients were included to account for potential dropouts. Statistical analysis was conducted using SPSS software (version 28.0, IBM Corp., Armonk, NY, USA), and figures were created using BioRender (https://www.biorender.com) and Figlinq (https://www.figlinq.com).

## Results

### Study Population

**Figure 1.**
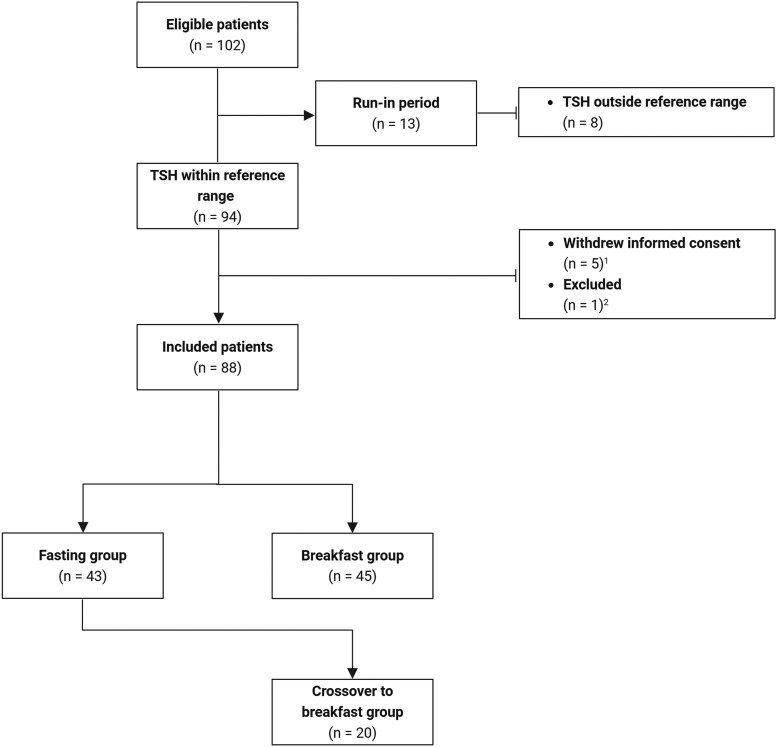
Flow chart of the study population. ^1^Reasons for withdrawal: other medical treatment not related to thyroid disease (n = 2), time commitment (n = 2), and anxiety related to TSH dysregulation (n = 1). ^2^Reason for exclusion: refusal to adjust levothyroxine dose (n = 1).

Between September 1, 2023, and January 24, 2025, 102 patients were deemed eligible for participation in this study. Of these, 8 were excluded because of persistent TSH levels outside the reference range during the run-in period, and 6 withdrew informed consent before randomization (Fig. 1). As such, 88 patients were included, of whom 90.9% ingested LT4 in a fasting state prior to the study. Patients were randomized into the fasting group (n = 43) or the breakfast group (n = 45). At baseline, there were no significant differences between groups in age, body mass index, hypothyroidism etiology, baseline TSH levels (Table 1), breakfast content (Table S2) (13), or use of interfering comedication. None of the patients used glucocorticoids.

**Table 1.** Baseline characteristics

	Fasting group (n = 43)	Breakfast group (n = 45)
Demographics		
Female sex at birth, n (%)	34 (79.1)	37 (82.2)
Age (years)	63 [47-69]	58 [49-70]
BMI (kg/m^2^)	27.4 [23.9-31.5]	28.1 [24.5-30.7]
Etiology of hypothyroidism, n (%)		
Primary hypothyroidism	24 (55.8)	25 (55.6)
Thyroidectomy or RAI (benign indications)	12 (27.9)	14 (31.1)
Thyroid cancer	5 (11.6)	3 (6.7)
Block and replace treatment for Graves’ disease	2 (4.7)	3 (6.7)
LT4 treatment		
Dose (mcg/kg)	1.3 [1.2-1.5]	1.4 [1.2-1.7]
Duration of treatment (year)	12 [3-20]	15 [3-19]
LT4 ingestion prior to study, n (%)		
≥ 30 minutes prebreakfast	39 (90.7)	41 (91.1)
Together with breakfast	4 (9.3)	4 (8.9)
LT4 brand, n (%)		
^©^Euthyrox	22 (51.2)	25 (55.6)
^©^Thyrofix	11 (25.6)	7 (15.6)
^©^Levothyroxinenatrium Teva	1 (2.3)	8 (17.8)
^©^Thyrax	4 (9.3)	3 (6.7)
^©^Tirosint (soft-gel capsule)	5 (11.6)	2 (4.4)
Laboratory analysis at baseline		
TSH (mIU/L)	1.03 [0.56-1.58]	1.35 [0.91-1.58]
FT4 (pmol/L)	19.9 [18.5-22.2]	19.9 [18.7-22.5]
TT3 (nmol/L)	1.3 [1.2-1.6]	1.4 [1.2-1.6]

Abbreviations: BMI, body mass index; FT4, free T4; LT4, levothyroxine; RAI, radioactive iodine; TT3, total T3.

Data presented as median [interquartile range] or n (%).

### Primary Outcome

**Figure 2.**
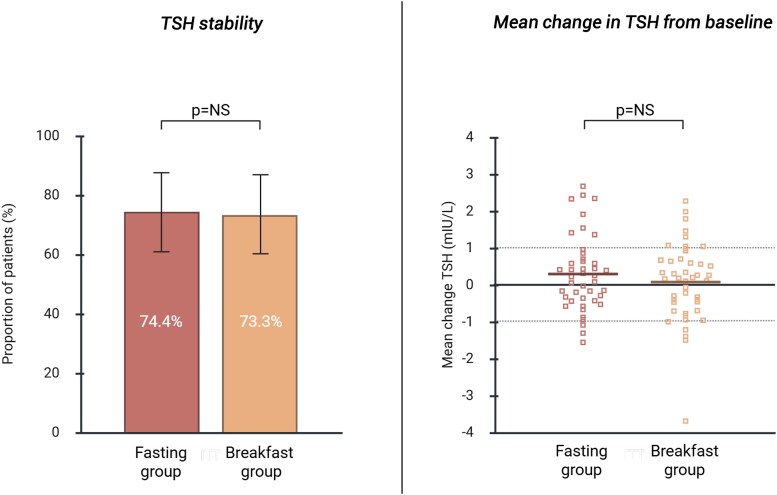
TSH stability and mean change in TSH levels from baseline. TSH stability was defined as meeting both of the following criteria: (1) two consecutive TSH measurements remaining within the reference range and (2) a maximum change of 1 mIU/L (increase or decrease) from baseline. Solid horizontal lines indicate mean changes in TSH levels. Dotted line indicates +/− 1 mIU/L change in TSH level from baseline. Abbreviation: NS, not significant.

The proportion of patients achieving TSH stability (defined as two consecutive TSH measurements remaining within the reference range and a change of < 1 mIU/L from baseline) was comparable between the fasting and the breakfast group (74.4% [95% confidence interval (CI): 61.0-88.0%] vs 73.3% [95% CI: 60.0-87.0%], *P* = not significant (NS), Fig. 2). TSH stability remained comparable after exclusion of relevant subgroups (eg, block-and-replace therapy for Graves’ disease, use of LT4 in soft-gel formulation) and was unaffected by breakfast composition; full sensitivity analyses are detailed in Tables S3 and S4 (13). Median time to TSH stability was similar in both groups (12 weeks). Mean change in TSH levels from baseline to study completion did not differ between the fasting and breakfast groups (absolute change in TSH: 0.27 ± 1.02 mIU/L and 0.29 ± 1.02 mIU/L respectively, *P* = NS, Fig. 2). Mean changes in FT4 and TT3 levels from baseline to study completion were comparable between both groups (Fig. 2 and Table S5) (13). The number and magnitude of dose adjustments at 6-, 12-, 18-, and 24-week follow-up visits for both groups are shown in Table S6 (13).

### Patient-reported Outcomes

**Figure 3.**
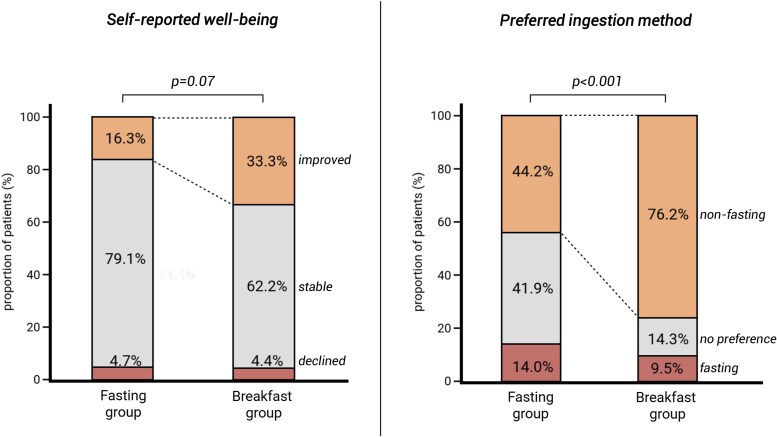
Patient-reported outcomes. *P-values represent difference in number of patients with improved self-reported well-being and preference for nonfasting levothyroxine ingestion between both groups.

Self-reported well-being improved in the breakfast group compared to the fasting group. In the breakfast group, 33.3% (n = 15) of patients reported an improvement in well-being, compared to 16.3% (n = 7) in the fasting group (*P* = .07, Fig. 3). Reasons for improved well-being in the breakfast group included feeling more physically fit (60.0%), psychological benefits of nonfasting ingestion (33.3%), relief from stomach discomfort after switching to nonfasting ingestion (26.7%), improved LT4 therapy adherence (13.3%), and a more consistent breakfast routine (6.7%). Furthermore, the breakfast group showed a stronger preference for taking LT4 with breakfast: 76.2% (n = 34) of patients in the breakfast group preferred nonfasting LT4 ingestion, compared to 44.2% (n = 19) in the fasting group (*P* < .001, Fig. 3). Moreover, 56 out of 63 (88.9%) patients in the breakfast group chose to continue nonfasting ingestion after the study. No adverse events were observed during the study.

### Crossover

**Figure 4.**
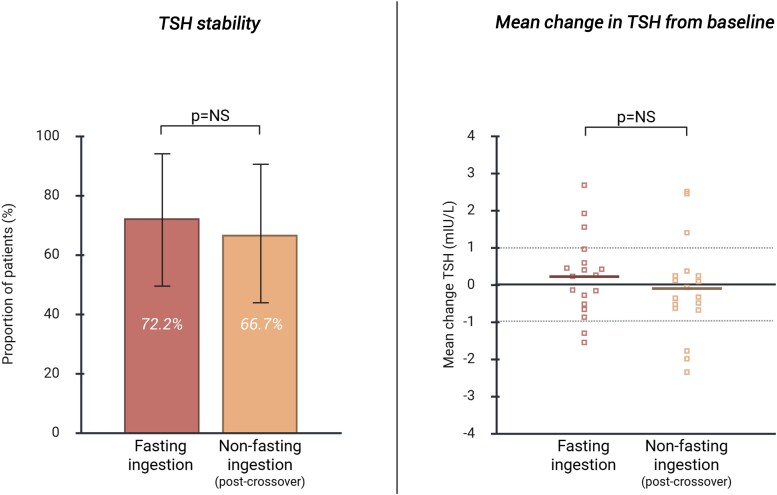
TSH stability and mean change in TSH levels from baseline in the crossover group. TSH stability was defined as meeting both of the following criteria: (1) two consecutive TSH measurements remaining within the reference range and (2) a maximum change of 1 mIU/L (increase or decrease) from baseline. Solid horizontal lines indicate mean changes in TSH levels. Dotted line indicates +/− 1 mIU/L change in TSH level from baseline. Abbreviation: NS, not significant.

Among the 32 patients in the fasting group who achieved TSH stability, 18 opted to cross over to the breakfast regimen. Reasons for opting out of crossing over are provided in Table S7 (13). Breakfast content remained consistent during both the fasting- and nonfasting regimens. Within the crossover group, TSH stability was achieved by 72.2% (95% CI: 49.0-95.0%) during the fasting regimen and 66.7% (95% CI: 43.0-91.0%) during the nonfasting regimen (*P* = NS, Fig. 4). Mean changes in TSH, FT4, and TT3 levels from baseline to study completion were comparable between both regimens (Fig. 4 and Table S5) (13). No significant change was observed in the median QoL composite scores on the ThyPro-39 questionnaire between fasting and nonfasting ingestion (Table S8) (13). Overall, 77.8% (n = 14) of the crossover patients preferred LT4 ingestion with breakfast over fasting ingestion.

## Discussion

In this randomized, proof-of-concept trial in patients with well-controlled hypothyroidism, 15% dose-adjusted LT4 ingestion with breakfast achieved a similar TSH stability compared to fasting LT4 ingestion. Self-reported well-being tended to be higher in the breakfast group, with 88.9% opting to continue breakfast ingestion after the study.

TSH levels remained stable with breakfast ingestion, with no significant difference in the change in TSH levels (0.27 ± 1.02 mIU/L vs 0.29 ± 1.02 mIU/L) between the breakfast and fasting groups, respectively. This between-group difference was smaller compared to +0.99 mIU/L and +1.99 mIU/L observed in similar studies with smaller sample sizes (6, 15). The reduced TSH variation observed likely resulted from the 15% LT4 dose increase in the breakfast group, which was intended to offset diminished absorption when LT4 is coingested with food (10).

Previous studies have shown that up to 40% of patients treated with LT4 had TSH levels outside the reference range (16, 17). Our study demonstrated more stable TSH levels, with 74.4% of patients in the fasting group and 73.3% in the breakfast group remaining within the reference range.

To our knowledge, no prior studies have investigated dose adjustments to counteract the decreased absorption by coingestion with breakfast. Our findings support that 15% dose-adjusted LT4 ingestion with breakfast is a viable and effective alternative to fasting ingestion. Patients who switched from fasting to breakfast ingestion reported improved well-being. Key benefits included a more consistent breakfast routine, improved drug adherence, relief from stomach discomfort, and both psychological and physical benefits (eg, feeling more physically fit). Similar improvements in QoL have been reported in a study where patients switched from fasting LT4 tablets to liquid LT4 taken with breakfast (8). Our previous questionnaire study of 410 patients treated with LT4 found that 25% regularly skipped breakfast and 13% forgot their medication due to fasting requirements, while 61% preferred nonfasting ingestion (5). In the current trial, 76.2% of patients in the breakfast group preferred nonfasting ingestion, with nearly 90% continuing this regimen after the study. These findings suggest that LT4 ingestion with breakfast could improve both patient well-being and medication adherence, reinforcing a strong patient preference for nonfasting LT4 ingestion.

This trial introduces a more convenient alternative to fasting LT4 ingestion, as the latter can interfere with patients’ routines and may compromise treatment adherence (9). In our previous study, 50% of patients reported being burdened by the required waiting time before breakfast (5). Alternative LT4 formulations, such as liquid and soft-gel preparations, are less dependent on gastric pH for absorption and can therefore be ingested with food, coffee, or interfering drugs (11, 12, 18). However, these formulations are not reimbursed universally and may not be suitable for all patients. Another alternative, bedtime LT4 ingestion (at least 3 hours after dinner), also maintains well-controlled hypothyroidism (19) but still requires prolonged fasting, which may be impractical for some. Since nonadherence and food or drug interactions contribute significantly to failure in achieving target TSH levels, the timing of LT4 ingestion should be individualized, with dose-adjusted ingestion at breakfast offering a practical and patient-preferred alternative.

This single-center study was limited by its relatively small sample size, which did not meet the threshold for a noninferiority trial. However, TSH stability rates were comparable between the fasting and breakfast groups, suggesting that a larger sample size would likely not have altered the overall conclusions. The randomized clinical trial design of the study strengthens the validity of the findings, and this trial represents the largest to date investigating alternative LT4 ingestion methods. The study population consisted of relatively well-controlled patients with hypothyroidism who were already receiving LT4 therapy. The limited sample size and relatively short follow-up duration precluded subgroup analyses, assessment of long-term effects, and conclusions regarding specific circumstances such as pregnancy, high-risk thyroid carcinoma, or heart failure. Future research should assess the effectiveness of nonfasting LT4 ingestion in newly diagnosed patients. Ideally, a large, multicenter trial with extended follow-up should be performed to evaluate the influence of different dietary components, various LT4 brands, potential subgroup differences (eg, by age, sex, or comorbidities), and the impact of nonfasting LT4 ingestion on quality of life.

In conclusion, this randomized proof-of-concept trial found that a 15% dose-adjusted LT4 regimen taken with breakfast maintained TSH stability similar to standard-dose fasting LT4 ingestion in patients with well-controlled hypothyroidism. Switching from fasting to nonfasting LT4 intake was associated with improved patient well-being and a strong patient preference for taking LT4 with breakfast. To enhance medication adherence, the timing of LT4 ingestion should be tailored to individual lifestyles, with dose-adjusted nonfasting ingestion offering a practical alternative.

## Data Availability

Some or all datasets generated during and/or analyzed during the current study are not publicly available but are available from the corresponding author on reasonable request.

## Clinical Trial Information

Trial Registration: Dutch National Registry (NL-OMON53296; https://onderzoekmetmensen.nl/nl/trial/53296).
