# Correction to: “Fasting vs Nonfasting, Dose-adjusted Levothyroxine Ingestion in Hypothyroidism: A Randomized Clinical Trial”

**DOI:** 10.1210/clinem/dgag020

**Published:** 2026-01-28

In the above-named article by Willems JIA, van Twist DJL, Helmich F, Sluiter T, Medici M, Peeters RP, Tummers-de Lind van Wijngaarden RFA (*J Clin Endo Metab*. 2025; doi: 10.1210/clinem/dgaf686), there was an error in a section heading.

In the originally published article, the section for author contributions had the heading “Author Xontributions.” The publisher regrets the error. In the corrected article, the section heading now reads “Author Contributions.”

The article has been corrected online.

doi: 10.1210/clinem/dgaf686

