# Correction to: “Fasting vs Nonfasting, Dose-adjusted Levothyroxine Ingestion in Hypothyroidism: A Randomized Clinical Trial”

**DOI:** 10.1210/clinem/dgag166

**Published:** 2026-04-29

In the above-named article by Willems JIA, van Twist DJL, Helmich F, Sluiter T, Medici M, Peeters RP, and Tummers-de Lind van Wijngaarden RFA (*J Clin Endo Metab.* 2026, 111(4), 938-944; doi: 10.1210/clinem/dgaf686), there was an error in the supplemental material reference:

In the originally published article, in Reference 13, there was an error in the supplemental material reference causing the file to not properly open in the repository due to have an expiration date: “Willems J, Van Twist D, Helmich F, *et al.* Supplemental data from: Fasting vs. non-fasting, dose-adjusted levothyroxine ingestion in hypothyroidism: a randomized clinical trial. *Figlinq*. Published online November 11, 2025. https://create.figlinq.com/∼j.willems/120.file.”

In the corrected article, the repository has been updated and Reference 13 reads: “Willems J, Van Twist D, Helmich F, *et al.* Supplemental data from: Fasting vs. non-fasting, dose-adjusted levothyroxine ingestion in hypothyroidism: a randomized clinical trial. *Zenodo.* Published online March 31, 2026. https://doi.org/10.5281/zenodo.19352514”

The article has been corrected online.

doi: 10.1210/clinem/dgaf686

